# Advances in supercritical fluid chromatography

**DOI:** 10.1002/ansa.202000163

**Published:** 2021-01-21

**Authors:** Lucie Nováková, Sebastiaan Eeltink

**Affiliations:** ^1^ Charles University Faculty of Pharmacy Department of Analytical Chemistry Prague Czech Republic; ^2^ Vrije Universiteit Brussel Department of Chemical Engineering Brussels Belgium

More than a century ago researchers recognized the unique properties that inorganic gases have above their critical point. Since then, different gases and liquids have been investigated. It was Ernst Klesper who reported on the very first chromatographic separations that used a supercritical fluid as the mobile phase back in 1962. In a brief report, he described the analysis of prophyrins using a polyethylene glycol stationary phases with dichlodifluoromethane and monochlorodifluoromethane at temperatures of 150–170°C at 55–160 bar, well above their critical points. It was however not until the 1980s that supercritical fluid chromatography (SFC) using commercial instrumentation emerged. During the same period, open‐tubular columns appeared as alternative to packed column technology, but since the 1990s packed‐column SFC is regaining popularity again. Another reason for the reemergence of SFC is for the fact that it is a green separation technology, as solvents are largely omitted and the main mobile phase constituent is carbon dioxide. CO_2_ is also cheap, nontoxic, and noncorrosive. The latter contributes to the robustness of the technology.

A supercritical fluid is obtained once the critical temperature and pressure for a substance are surpassed. Above this point, the intermolecular van der Waals forces are affected such that molecules form a distinct phase, providing unique properties in solubility. Furthermore, its properties, such as density, viscosity, and diffusion coefficient vary with temperature and operating pressure. Consequently, modifier content, and also pressure and temperature, can be adjusted to establish unique selectivity in SFC. As diffusion coefficients are significantly higher than that in HPLC, the resistance to mass transfer is much lower. This means that experiments can be performed at significantly higher mobile‐phase velocities, allowing to increase the throughput without the loss of separation efficiency.

The benefits of SFC are already recognized for a variety of application areas, including the analysis of analytes unsoluble in aqueous environment, such as lipids, polymers, and fuels. SFC is now well established in the field of the pharmaceutical industry, especially for the analysis of chiral compounds, both on analytical and preparative scales. To proliferate SFC further, good insights are required in the fundamentals of SFC. Furthermore, with this special issue we aim to show the potential and benefits, but also its limitations. Finally, this special issue includes a number of review papers, discussing the current state of the art and highlighting new application possibilities.

As the guest editors of this special issue, we would like to acknowledge all the authors for their contributions. Last, but not least, a special thank belongs the reviewers providing valuable input and advices.

